# SWI/SNF Component BAF250a Coordinates OCT4 and WNT Signaling Pathway to Control Cardiac Lineage Differentiation

**DOI:** 10.3389/fcell.2019.00358

**Published:** 2020-01-22

**Authors:** Ienglam Lei, Shuo Tian, Victor Chen, Yong Zhao, Zhong Wang

**Affiliations:** ^1^Faculty of Health Sciences, University of Macau, Taipa, China; ^2^Department of Cardiac Surgery, Cardiovascular Center, University of Michigan, Ann Arbor, MI, United States; ^3^Henan Provincial People’s Hospital, Fuwai Central China Cardiovascular Hospital, Key Laboratory of Cardiac Regenerative Medicine, National Health and Family Planning Commission, Central China Branch of National Center for Cardiovascular Diseases Henan Province, Zhengzhou, China

**Keywords:** SWI/SNF complex, cardiac differentiation, epigenetics, cell cycle, BAF250a, human ESCs, cardiomyocyte differentiation, OCT4

## Abstract

Dissecting epigenetic mechanisms controlling early cardiac differentiation will provide insights into heart regeneration and heart disease treatment. SWI/SNF complexes remodel nucleosomes to regulate gene expression and play a key role in organogenesis. Here, we reported a unique function of BAF250a in regulating the physical interaction of OCT4 and β-CATENIN during cardiac lineage differentiation from human ESCs. BAF250a deletion greatly reduced the physical interaction between OCT4 and β-CATENIN but did not alter the expression of β-CATENIN and OCT4 in the mesodermal progenitor cells. BAF250a ablation led to decreased recruitment of OCT4 and β-CATENIN at promoters of key mesodermal lineage genes, such as MESP1 and EOMES. Subsequently, the expression of lineage-specific genes was downregulated, whereas the expression of pluripotent genes was upregulated. In parallel, BAF250a ablation also altered recruitments of OCT4 and β-CATENIN to the promoter of CCND2 and CCND3, two key genes for S phase entry during cell cycle. Consequently, BAF250a deletion led to prolonged S phase in Mesp1^+^ cardiac progenitor cells, which in turn inhibited efficient differentiation of Mesp1^+^ to Isl1^+^ cells. Furthermore, BAF250a deletion abolished the interaction of OCT4 and BRG1 in mesoderm, suggesting that BAF250a is the key component in SWI/SNF complex that determines the interaction of Oct4/β-catenin in mesoderm. In contrast, we found that BAF250a did not regulate the OCT4/β-CATENIN interaction during neuroectoderm differentiation. Altogether, our results suggest that BAF250a specifically controls proper cardiac mesoderm differentiation by reorganizing the binding of OCT4/β-CATENIN and regulates both key lineage differentiation genes and cell cycle genes that coincided in response to WNT/β-CATENIN signal.

## Introduction

Dissecting the molecular mechanisms underlying the differentiation of pluripotent stem cells (PSCs) into tissue-specific progenitors and terminal differentiated cell types is key to understanding organ development and regeneration. Within heart, embryonic cardiac progenitor cells (CPCs) are promising cell sources for cell-based therapies for heart disease. Direct differentiation of CPCs from PSCs has been achieved based on the knowledge of embryonic development (Kattman et al., 2011; Lian et al., 2012). Each step of differentiation is tightly regulated by stage-specific signals and epigenetic regulation (Alexander et al., 2015). In particular, an initial WNT activation and subsequent WNT inhibition is required for CPC differentiation (Burridge et al., 2014). In parallel, epigenetic regulations including histone modification and chromatin remodeling are also believed to contribute to the precise of cardiac differentiation (Wamstad et al., 2012). However, how WNT signaling and epigenetic regulation work together to direct cardiac differentiation is poorly understood.

SWI/SNF ATP-dependent chromatin remodeling complex modulates the chromatin accessibility by reorganizing nucleosomes to regulate chromatin accessibility and are essential in organogenesis (Ho and Crabtree, 2010). BAF complexes also interact directly with other epigenetic factors and transcription factors in regulating gene expression. Studies from numerous research groups including us reveal that BAF complexes are important for embryonic stem cell (ESC) pluripotency and cardiogenesis (Ho et al., 2011; Lei et al., 2012, 2013; Wu et al., 2014; Lei I. et al., 2015). Importantly, BAF complex interacts with pluripotent genes, such as OCT4 and SOX2, to regulate epigenetic landscape in ES cells and during ES differentiation (Ho et al., 2009; King and Klose, 2017). In particular, both OCT4 and BAF localize at the bivalent domains in ES cells, suggesting potential synergistic roles of OCT4/BAF complexes during differentiation. Nevertheless, the underlying epigenetic mechanisms of OCT4/BAF-mediated cardiac lineage differentiation in response to extrinsic signals are not well understood.

Cell cycle control is another key regulatory process during differentiation. Recent studies have identified cross-talk between cell cycle and cell fate determination of human ESCs (hESCs) (Pauklin and Vallier, 2013; Dalton, 2015). The hESCs have a unique cell cycle pattern with a short G1 phase, while their differentiation lengthened the G1 phase (Calder et al., 2013). The differentiation potential of hESCs varies at different cell cycle stage. It is shown that the activity of Activin/Nodal signal is regulated by cyclinD-CDK4/6 complex in early and late G1 stage (Pauklin and Vallier, 2013). S and G2 phases have further been found to actively promote pluripotent state (Gonzales et al., 2015). These studies suggest that cell cycle phases could play an essential role in commitment of mesodermal cells into cardiac lineages, a notion that remains to be confirmed.

In this study, we define a unique function of BAF in regulating cardiac lineage differentiation in human ESCs by examining a key regulatory subunit BAF250a. By taking advantage of conditional BAF250a knockout human ESCs we generated (Bu et al., 2010), we observed that BAF250a is required for the physical interaction of OCT4 and β-CATENIN in mesoderm cells. Consequently, BAF250a deletion affected the recruitment of OCT4 and β-CATENIN at target gene promoters and altered gene expression of differentiation, pluripotent, and cell cycle genes. We further revealed that the BAF250a deletion led to S phase population increase of mesodermal cells, which have less differential potential into Isl1^+^ CPC cells. In addition, we found that BAF250a was dispensable for the interaction of OCT4 and β-CATENIN during neuroectoderm differentiation and BAF250a deficiency did not affect neuroectoderm differentiation. These results suggest an important role of BAF250a in reorganizing the binding of OCT4/β-CATENIN and regulating both key genes for lineage differentiation and cell cycle phases for proper cardiac mesoderm differentiation in response to WNT signal.

## Materials and Methods

### Human ESCs Culture and Differentiation

Human ESCs were maintained on Matrigel-coated dishes with mTseR1 medium as described (Lei I.L. et al., 2015). The differentiation of hESC to cardiomyocyte was performed using sequential WNT modulation (Burridge et al., 2014). Briefly, hESCs were dissociated into single cells with Versene solution. One million hESCs were plated to a 35-mm dish with 10 μM Rock inhibitor. Six micromolar GSK3 inhibitor CHIR99021 in chemical defined medium (CDM, 0.5% Human Albumin in RPMI1640) was added to the confluent hESC to induce the differentiation for 2 days. Cells were recovered for 2 days in CDM, and then treated with 2 μM IWR1 in CDM for another 2 days. The culture medium was changed to CDM for 2 days, and then 3% knockout serum replacement was added to CDM in subsequent cultures. For neuroectoderm differentiation, the hESC monolayer culture was subjected to differentiation medium (20% KOSR in KO DMEM) containing 500 nM LDN193189 and 10 μM SB431542 for 4 days (Tchieu et al., 2017). Then, the differentiation medium was changed to contain 25%, 50%, 75%, and 100% N2 medium every other day from day 4 to day 12. The medium was changed every day. Day 12 cells were harvested for FACS and RT-qPCR analyses. For FACS analysis, the antibodies used against Isl1 (DSHB,39.4D5, 1:100), MESP1 (Aviva systems biology, ARP39374_P050, 1:100), PAX6 (R&D, AF8150, 1:200), and cTnT (Thermo Fisher, MS295, 1:250), as well as normal IgG (CST, 2729S, 1:100), were incubated with cells from specific stage at 4°C overnight, washed three times with PBS, and followed with half an hour incubation of secondary antibody. After washing three times, the percentage of stained cells were analyzed by FACS.

### Western Blot

Cells were lysed in 0.25% Triton X-100, 50 mM Tris–HCl (pH 7.4), 150 mM NaCl, and 1 × protease inhibitors (Roche) and then spun at 16000 × *g* for 5 min at 4°C. Laemmli sample buffer was added before boiling for 5 min. Primary antibody OCT4 (sc-8628, 1:1000), BRG1 (ab4081, 1:1000), BAF250a (sc-32761, 1:1000), T (sc-17745, 1:1000, 1:100 for staining), active β-CATENIN (CST 4270, 1:1000), and total β-ACTIN (CST4970, 1:2000) were applied to the blots at 4°C overnight. Secondary antibody from LiCOR was used for detection.

### Co-immunoprecipitation

The co-immunoprecipitation was performed as previously described (Lei I. et al., 2015). Briefly, day 1 differentiated cells were harvested in cold PBS and extracted for 30 min at lysis buffer (20 mM HEPES, pH 8.0, 150 mM NaCl, 1% NP-40, and 2 mM EDTA) with proteinase inhibitors. After centrifugation, 5% of supernatant was kept in 4°C as input. The remaining supernatant was separated into equal volume and incubated with 1 μg of OCT4 or rabbit IgG with 20 μl of Dynabeads at 4°C overnight. The complex was washed four times in wash buffer (20 mM HEPES, pH 8.0, 150 mM NaCl, 0.1% NP-40, and 2 mM EDTA). Beads were boiled for 5 min in 1 × SDS buffer to denature proteins. After SDS-PAGE, Western blots were performed using antibody against β-CATENIN (CST8480, 1:1000) and active β-CATENIN (1:1000).

### Immunostaining

Embryos were fixed in 4% paraformaldehyde at 4°C overnight and embedded in paraffin. Sections were collected using microtome. Sections were blocked with 10% horse serum in PBS buffer containing 0.1% Tween 20 and 0.1% Triton X-100 for 1 h at room temperature and incubated with goat anti-T (sc-17745 1:100), mouse anti-OCT4 (sc-8628, 1:200), and rabbit active β-CATENIN (CST 4270, 1:200) at 37°C for 1 h. Then, sections were washed three times with PBS buffer with 0.1% Tween 20 before being incubated with a donkey anti-goat Alexa 488-conjugated, donkey anti-mouse Alexa 594-conjugated, and donkey anti-rabbit Alexa 647-conjugated antibodies at room temperature for 1 h. After washes, section slides were mounted and analyzed by fluorescent microscopy.

### Proximity Ligation Assay (PLA)

Proximity ligation assay was performed using Duolink^®^ *In Situ* Red kit (DUO92101) according to Sigma’s protocol. Briefly, E7 embryo sections were dewaxed with xylene, and rehydrate with descending alcohol. Antigen retrieval was performed by microwaving section in pH 9 Tris–EDTA buffer for 15 min. The section was permeabilized with 0.2% Triton X-100 and blocked in 5% horse serum for 1 h. Incubation with primary antibodies OCT4 and β-CATENIN was performed at 4°C overnight. Sections were washed three times in PBST. The ligation and detection of OCT4/β-CATENIN by proximity probes were performed according the manufacturer’s protocol.

### Chromatin Immunoprecipitation

ChIP experiments were performed as previously described (Lei I. et al., 2015). Briefly, cells were fixed in 1% formaldehyde for 10 min and quenched with 0.125 M glycine for 5 min. Cells were then harvested and sonicated into 200–1000 bp using a Branson Sonifier. Chromatin solution was incubated with Dynabeads and antibodies against OCT4 (sc-8628), β-CATENIN (CST 4270), H3K27me3 (Millipore 07-449), BRG1 (Abcam, ab70558), and SUZ12 (Abcam, ab12073) overnight. Beads were washed four times with LiCl wash buffer followed by one wash of TE buffer then eluted with elution buffer at 65°C. DNA was purified using phenol:chloroform extraction. Enrichment of immunoprecipitated DNA was then validated by quantitative PCR.

## Results

### BAF250a Deletion Disrupted Cardiomyocyte Differentiation but Not Primitive Induction of Human ESCs

**FIGURE 1:**
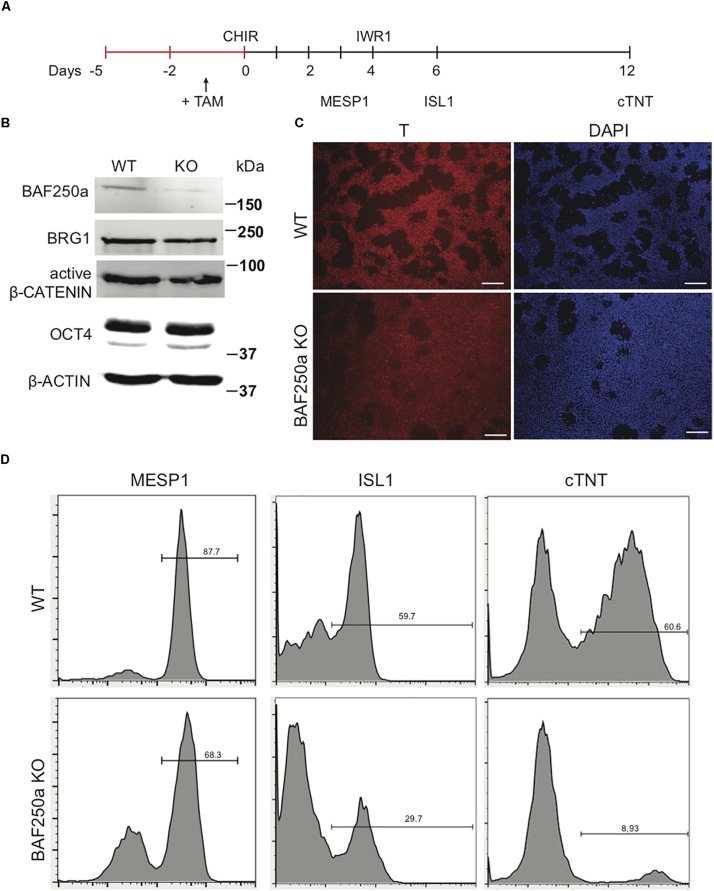
BAF250a deletion in hESCs led to cardiac differentiation defects. **(A)** Schematic diagram of cardiac differentiation. **(B)** Western blot of BAF250a, BRG1, active β-CATENIN, and OCT4 in WT and BAF250a KO cells at differentiation day 1. β-actin was used as a loading control. **(C)** Immunostaining of T at differentiation day 1 in both WT and BAF250a KO cells. **(D)** FACS analysis of differentiation of WT and BAF250a KO hESC to mesoderm, cardiac progenitors, and cardiomyocytes using MESP1, ISL1, and cTNT antibodies at differentiation day 3, 6, and 10. Scale bar: 50 μm.

To examine the role of BAF250a in human cardiac differentiation, we used direct differentiation protocol (Figure 1A) in a 4-hydroxytamoxifen (4-OHT)-inducible BAF250a knockout (KO) hESC line we generated (Bu et al., 2010; Burridge et al., 2014). 4-OHT was added 1 day before induction of differentiation (day - 1). At differentiation day 1, the expression of BAF250a was significantly abolished with 4-OHT treatment, whereas the expression of the ATPase subunit Brg1 was not changed. In addition, the expression levels of key regulator in mesoderm differentiation such as active β-CATENIN and OCT4 did not show significant changes at day 1 (Figure 1B). Moreover, the percentage of T^+^ cell (mesoendoderm) was not affected after BAF250a deletion (Figure 1C). In contrast, deletion of BAF250a at ESC stage led to decreased differentiation efficiency of cardiac mesoderm (MESP1^+^) cell and ISL1^+^ CPCs (Figure 1D) and significant reduction of cTNT^+^ CMs (Figure 1D). These results indicated that BAF250a was dispensable in the primitive induction during hESC differentiation and was required for efficient cardiomyocyte differentiation from mesodermal progenitor cells.

### BAF250a Was Required for OCT4/β-CATENIN Interaction

**FIGURE 2:**
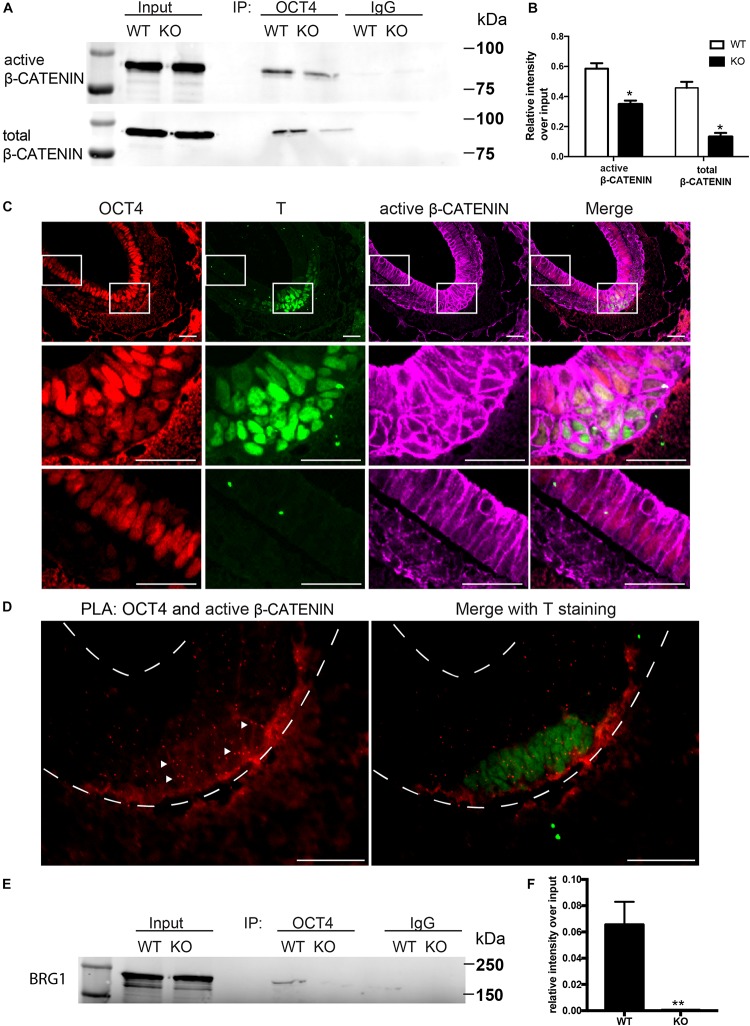
BAF250a regulated proper OCT4 and β-CATENIN interaction during differentiation. **(A)** Immunoprecipitation of day 1 differentiation mesodermal cells with anti-OCT4 antibody, followed by Western blotting to detect β-CATENIN and active β-CATENIN. Five percent of cell lysates were used as input controls. **(B)** Relative band intensity of precipitated β-CATENIN and active β-CATENIN in WT and BAF250 KO cells. **(C)** Immunostaining of OCT4, T, and active β-CATENIN showing their colocalization at E7.0 embryos. Regions in white box were shown with higher magnification in the second and third row. **(D)** OCT4 and active β-CATENIN interaction in E7.0 embryos detected by PLA. Scale bar: 50 μm. **(E)** Immunoprecipitation of day 1 differentiation mesodermal cells using anti-OCT4 antibody, followed by Western blotting using BRG1 antibody to detect BRG1. Five percent of cell lysates were used as input controls. **(F)** Relative band intensity of precipitated BRG1 in WT and BAF250 KO cells. *p < 0.05, **p < 0.01, *n* = 3.

WNT/β-CATENIN signaling pathway is essential for gastrulation and mesodermal formation and are key factors in the established cardiac lineage differentiation from hESCs (Lian et al., 2013). We therefore examined the expression and activity of WNT/β-CATENIN with and without BAF250a during hESC differentiation. We found that the expression of active form β-CATENIN in day 1 differentiated cells was not changed after BAF250a deletion (Figure 1C). Moreover, the expression of OCT4, which has been shown to have important roles in mesoderm differentiation (Funa et al., 2015; Wen et al., 2017), was not affected by BAF250a deletion (Figure 1C). As BAF250a could interact with OCT4 to regulate cell fate (Ding et al., 2012) and OCT4/β-CATENIN interaction is important for proper mesoderm differentiation (Funa et al., 2015), we hypothesized that BAF250a might be required for the physical interaction of OCT4 and β-CATENIN in the mesoendoderm cells during hESC differentiation. Co-immunoprecipitation experiments at day 1 of differentiation demonstrated decreased interaction between OCT4 and β-CATENIN or active β-CATENIN in BAF250a-depleted cells (Figures 2A,B), indicating that BAF250a was required for efficiency physical interaction of OCT4 and β-CATENIN. Consistent with this finding, we detected that OCT4 and β-CATENIN was colocalized at T^+^ cells at E7.0 mouse embryos (Figure 2C). β-CATENIN was also highly expressed in the nucleus in the colocalized cell compared to OCT4 only expressed cells (Figure 2C). Moreover, enriched OCT4 and β-CATENIN interaction signals were also observed in T^+^ cells in E7.0 embryos by PLA (Leuchowius et al., 2011; Figure 2D). As BRG1, the core catalytic subunit of SWI/SNF complex, has been shown to interact with OCT4 in ESCs (van den Berg et al., 2010) and BAF250a is considered a regulatory subunit and does not affect the formation of the core SWI/SNF complex, we wonder whether the observed BAF250a and OCT4/β-CATENIN interaction is secondary to BRG1 and OCT4/β-CATENIN interaction. Surprisingly, we found that the interaction of OCT4 and BRG1 was abolished in BAF250a KO cells (Figures 2E,F). Since BRG1 directly interacts with β-CATENIN (Barker et al., 2001), our results suggested that BAF250a/BAF acted as a physical mediator to regulate OCT4/β-CATENIN interaction.

### Proper OCT4 and β-CATENIN Interaction Mediated by BAF250a Guided Cardiac Mesoderm Gene Expression

**FIGURE 3:**
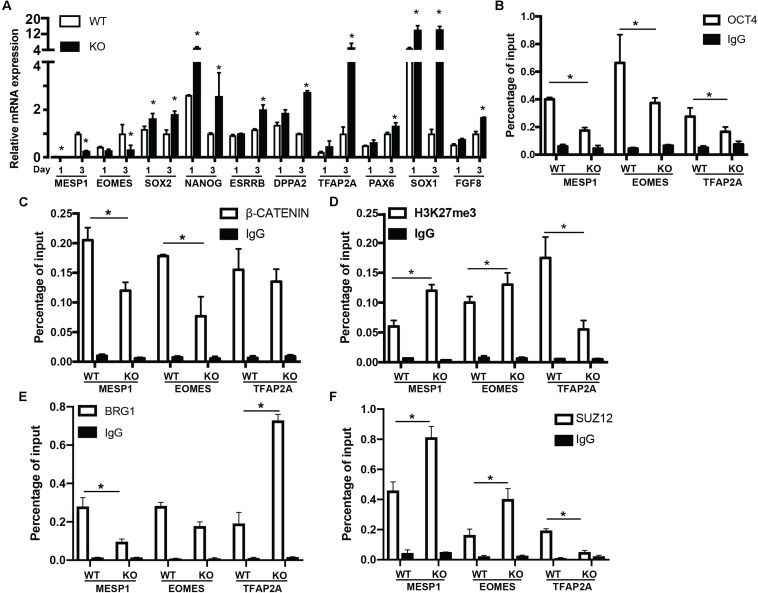
BAF250a controlled cardiac mesoderm gene expression. **(A)** Expression of MESP1, EOMES, SOX2, NANOG, ESRRB, DPPA2, TFAP2A, PAX6, SOX1, and FGF8 at differentiation day 1 and day 3. ChIP assays with **(B)** anti-OCT4, **(C)** anti-β-CATENIN, **(D)** anti-H3K27me3, **(E)** anti-BRG1, and **(F)** anti-SUZ12 antibodies using day 1 differentiated cells at MESP1, EOMES, and TFAP2A promoters. *p < 0.05, *n* = 3.

To reveal the function of BAF250a-mediated OCT4/β-CATENIN interaction in the differentiation of cardiac mesoderm, we first investigated the mesodermal gene expression at day 1 and day 3 after hESC differentiation. MESP1 and EOMES activation was repressed in BAF250a KO cells at day 3 of differentiation. We also found that the expression of pluripotent genes SOX2, NANOG, ESRRB, and DPPA2 was not properly turned down in BAF250a KO cells. In addition, we also detected upregulation of neural gene expression, such as TFAP2A, PAX6, SOX1, and FGF8 (Figure 3A). These results suggest that BAF250a is required for proper silencing of pluripotent genes and neural genes during differentiation. Given that the SWI/SNF complex is essential to create accessible chromatins (King and Klose, 2017), we then examined the recruitment of OCT4 and β-CATENIN at MESP1, EOMES, and TFAP2A promoters, as OCT4/β-CATENIN interaction is important for the activation of cardiac mesoderm genes and repressing neural genes. ChIP assays showed decreased recruitment of OCT4 and β-CATENIN to MESP1 and EOMES promoters, whereas only decrease of OCT4 recruitment was found at TFAP2A promoters (Figures 3B,C). To determine whether the disrupted recruitment of OCT4 and β-CATENIN is important for mesodermal gene activation, we examined H3K27me3 changes at MESP1 and EOMES promoters. We found that the H3K27me3 modifications at MESP1 and EOMES promoters were not efficiently removed in BAF250a KO cells, while a lower H3K27me3 level was found in TFAP2A promoters (Figure 3D). Moreover, we found that BRG1 recruitment to the promoters of MESP1 and EOMES was reduced in BAF250a KO cells (Figure 3E). Consistent with the previous finding that BRG1 evicts the SUZ12 recruitment for subsequent H3K27me3 regulation (Lei I. et al., 2015; Kadoch et al., 2017), we found that SUZ12 recruitment was increased at the promoters of MESP1 and EOMES but reduced at the TFAP2 promoter (Figure 3F). Thus, our results suggested that BAF250a-mediated OCT4/β-CATENIN interaction was required for their efficient recruitment to mesoderm targets and subsequent epigenetic remodeling.

**FIGURE 4:**
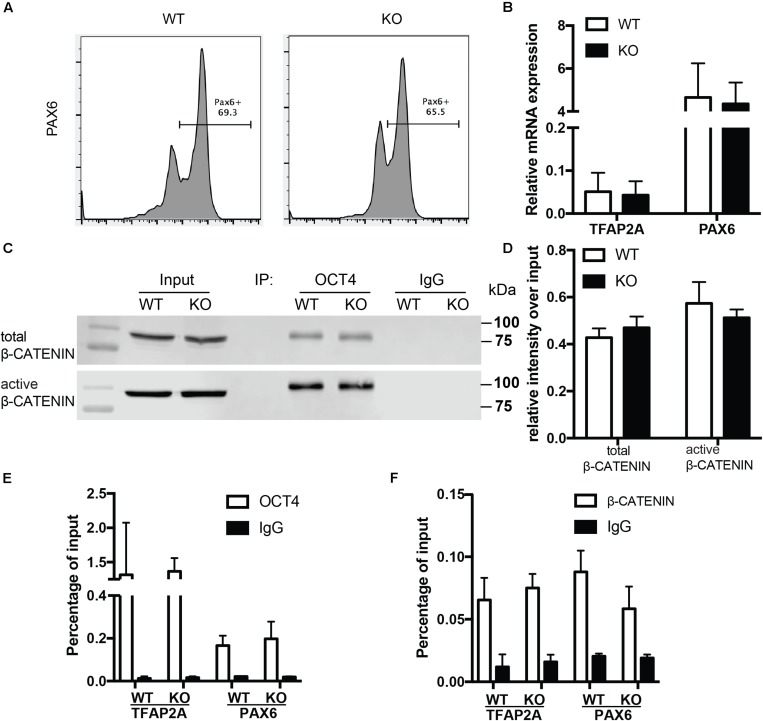
BAF250a was dispensable for neuroectoderm differentiation. **(A)** FACS analysis of differentiation of WT and BAF250a KO hESC to neuroectoderm using PAX6 antibody at differentiation day 12. **(B)** Relative mRNA expression of TFAP2A and PAX6 at day 12 differentiation. **(C)** Immunoprecipitation of day 2 differentiation cells after LDN193189 and SB43142 treatment using anti-OCT4 antibody, followed by Western blotting to detect β-CATENIN and active β-CATENIN. Five percent of cell lysates were used as input controls. **(D)** Relative band intensity of precipitated β-CATENIN and active β-CATENIN in WT and BAF250 KO cells. ChIP assays with **(E)** anti-OCT4 and **(F)** anti-β-CATENIN antibodies using day 2 differentiated cells after LDN193189 and SB43142 treatment at TFAP2A and PAX6 promoters. *n* = 3.

To examine whether the role of BAF250a is specific for mesoderm lineage, we next determined the differentiation efficiency of neuroectoderm from hESC using a neuroectoderm differentiation protocol with dual inhibition of BMP and TGFβ signaling (Tchieu et al., 2017) using both WT and BAF250a KO hESCs. We found that the percentage of Pax6^+^ neuroectoderm cells was not affected in BAF250a KO cells at day 12 after inducing differentiation (Figure 4A). The expression levels of TFAP2A and PAX6 were also comparable between WT and BAF250a KO cells (Figure 4B). Importantly, we found that the interaction of OCT4 and β-CATENIN was not affected in BAF250a KO cells at day 2 of neuroectorderm differentiation (Figures 4C,D). Furthermore, the recruitment of OCT4 and β-CATENIN to TFAP2A and PAX6 was not changed in BAF250a KO cells (Figures 4E,F). These results suggested that BAF250a was dispensable for the interaction of OCT4 and β-CATENIN during neuroectoderm differentiation, which was distinct from its key role in regulating the interaction of OCT4 and β-CATENIN during mesoderm differentiation.

### BAF250a Deletion Led to Prolonged S-Phase in MESP1^+^ Cells and Poor Differentiation Potential of MESP1^+^ Cells to ISL1^+^ CPCs

**FIGURE 5:**
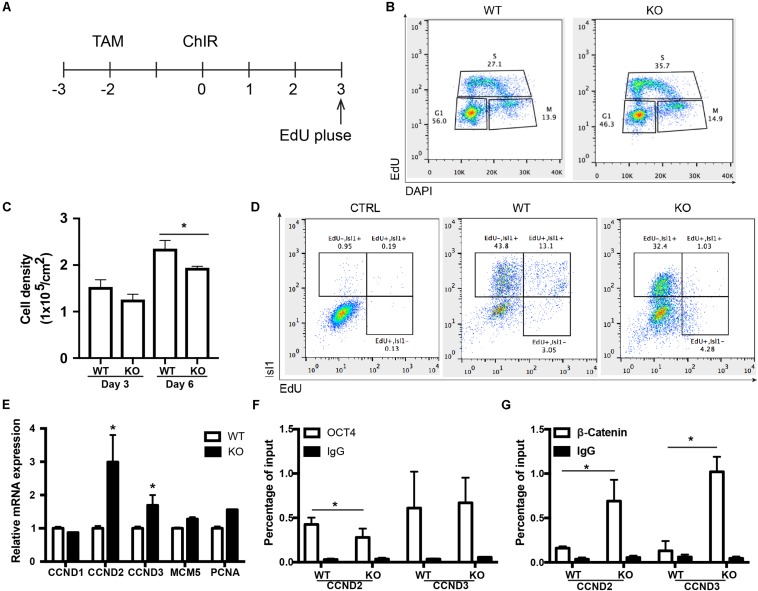
BAF250a regulated the cell cycle of MESP1 cells and promoted MESP1 cell differentiation to Isl1 CPCs. **(A)** Schematic diagram of EdU labeling. **(B)** Representative images of cell cycle analysis at differentiation day 3 with EdU and DAPI. **(C)** Cell density in day 3 and 6 of differentiation in WT and BAF250a KO cells. **(D)** Representative images of FACS analysis of Isl1^+^ cells at differentiation day 6 using EdU pulse labeling. **(E)** Expression of cell cycle genes CCND1, CCND2, CCND3, MCM5, and PCNA at differentiation day 3. ChIP assays with **(F)** anti-OCT4 and **(G)** anti-β-CATENIN antibodies using day 3 differentiated cells. *n* = 3. *p < 0.05 vs. WT.

To further determine how the disassociation of OCT4/ β-CATENIN contributed to the later cardiac differentiation defects shown in Figure 1, we examined how the cell cycle and genes essential for cell cycle regulation were affected by BAF250a ablation, as cell cycle control is a key regulatory process for differentiation (Ruijtenberg and van den Heuvel, 2015), and BAF250a, OCT4, and WNT/β-CATENIN are all involved in cell cycle regulation. We labeled the proliferating mesoderm cells with 5-ethynyl-2-deoxyuridine (EdU) pulse at day 3 for 1 h (Figure 5A). FACS analysis was performed immediately after EdU pulse labeling and showed a higher percentage of S phase cell population and a lower G1 phase cell population in BAF250a-deficient cells (Figure 5B). Moreover, BAF250a KO cells grew slower than WT cells during differentiation days 3 and 6 (Figure 5C). Thus, our results suggested that BAF250a deletion led to prolonged S-phase in MESP1^+^ cells. When we examined the EdU-labeled cells at Isl1 CPC stage, we detected about 43.8% ± 7.4% of EdU^–^/ISL1^+^ cells and 13.0% ± 4.3% of EdU^+^/ISL1^+^ cells from WT hESC differentiation, but only 32.4% ± 5.6% of EdU^–^/ISL1^+^ cells (*p* = 0.05) and 1.0% ± 0.4% Edu^+^/ISL1^+^ cells (*p* = 0.04) from the differentiation of BAF250a KO hESCs (Figure 5D), suggesting that the ISL1^+^ cells are largely derived from non-proliferating mesoderm cells and BAF250a could contribute to the differentiation of ISL1^+^ cells by regulating cell cycle of mesoderm cells.

We next examined the cell cycle genes regulated by BAF250a, together with OCT4 and WNT/β-CATENIN. We found that expression of G1/S transition protein CCND2 and CCND3 was significantly higher in BAF250a KO cells (Figure 5E). Furthermore, there was decreased recruitment of OCT4 and increased recruitment of β-CATENIN at both CCND2 and CCND3 promoters (Figures 5F,G). Together with the interaction of OCT4 and β-CATENIN in efficient recruitment of β-CATENIN to mesoderm gene promoters (Figures 3B,C), our results suggested that interaction of β-catenin with OCT4 decreases the recruitment of β-catenin on CCNDs and increased the recruitment of β-catenin to genes for mesoderm differentiation, thereby enhancing cardiac differentiation.

## Discussion

In this study, we show that BAF250a regulates cardiac lineage specification through regulating the interaction of OCT4 and β-CATENIN and their recruitment to target genes. BAF250a-mediated OCT4/β-CATENIN interaction not only fine-tunes the MESP1 and EOMES gene expression but also regulates the cell cycle progression of mesoderm cells. We further demonstrate that BAF250a regulates G1/S transition in mesoderm cells, which is critical for the differentiation into cardiac lineage. Therefore, by modulating the interaction and recruitment of OCT4 and β-CATENIN, BAF250a guides the direct cell-type-specific gene expression and the cell cycle machinery in mesodermal cells to promote cardiac lineage commitment. Importantly, our data indicate that BAF250a is a key subunit regulating SWI/SNF activity. We show that BAF250a is required for BRG1 interaction with OCT4 in mesoderm cells. In addition, the observed key role of BAF250a in regulating cardiac mesoderm differentiation appears lineage specific, as BAF250a is dispensable for the interaction of OCT4 and β-CATENIN during neuroectoderm differentiation and BAF250a deficiency does not affect neuroectoderm differentiation.

We report here that BAF250a is required for efficient interaction of OCT4 and β-CATENIN in T^+^ cells for proper cardiac mesodermal differentiation. Direct differentiation of cardiomyocytes from human ESCs is based on sequential stimulation and inhibition of WNT/β-CATENIN signals identified during cardiogenesis (Burridge et al., 2014). Dynamic Oct4 recruitment to the genome in response to different signals is required for proper differentiation of mesoderm from hESCs (Funa et al., 2015; Tsankov et al., 2015) and the interaction of OCT4 and β-CATENIN is essential for mesoderm differentiation (Funa et al., 2015). In this report, we have identified that the physical interaction of OCT4 and β-CATENIN is significantly reduced in BAF250a KO cells, which leads to aberrant recruitment of Oct4 and β-CATENIN to the promoter of mesoderm genes, such as MESP1 and EOMES. OCT4/β-CATENIN has been revealed to prepattern the epigenome by regulating PRC2 recruitment to the mesoendoderm genes, whereas BAF250a/BAF complex is required for PRC2 eviction in the genome (Kadoch et al., 2017). In our study, we find reduced recruitment and subsequent increase of H3K27me3 at MESP1 and EOMES promoters during differentiation in BAF250a KO cells. Based on these studies and our findings presented here, BAF250a-mediated OCT4/β-CATENIN interaction likely plays an important role in efficiently removing mesoderm differentiation barrier during differentiation.

Indeed, our studies show that the aberrant recruitment of Oct4 and β-CATENIN leads to altered expression of several key groups of genes that negatively affect cardiac lineage differentiation. Particularly, the expression of key mesodermal lineage gene MESP1 and EOMES is decreased. In contrast, we have identified upregulation of pluripotent genes, such as SOX2, NANOG, ESRRB, and DPPA2, in BAF250a knockout cells during differentiation. Our finding is consistent with reported studies that the interaction of OCT4 and β-CATENIN is essential for the repression of SOX2 and activation of mesoderm genes (Funa et al., 2015; Ying et al., 2015; Rao et al., 2016). Importantly, the upregulation of neural gene such as TFAP2A we find in BAF250a KO cells could be a direct consequence of OCT4 and β-CATENIN complex dissociation, because β-CATENIN induces the expression of neural genes in the absence of OCT4 (Funa et al., 2015). Furthermore, we find that BAF250a deletion does not alter the interaction and the recruitment of OCT4/β-CATENIN during neuroectoderm differentiation. These results suggest that BAF250a is a key subunit defining the specificity of SWI/SNF in mesoderm cells.

Our findings on the connection of cell cycle regulation and direct differentiation in mesodermal cells highlight the importance of cell cycle regulation in direct differentiation in the multipotent cells. We find that BAF250a regulates the cell cycle of mesoderm cell through controlling the expression of cell cycle gene CCND2/3. This is consistent with previous studies that BAF complexes could act as transcriptional repressor of positive cell cycle factors in G1/S transitions (Ruijtenberg and van den Heuvel, 2015). As Cyclin D proteins have been suggested to modulate the transcriptional activity of SMAD2/3 to promote neuroectoderm differentiation of hESCs (Pauklin and Vallier, 2013), it is likely that BAF250a-mediated OCT4/β-CATENIN complex could directly activate cardiac gene program while also mediating cell cycle exit. Indeed, our finding that most of Isl1^+^ cells are derived from non-proliferating mesoderm cells reveals a significant role of cell cycle in regulating cell differentiation. Importantly, BAF250a deletion reduces the cardiac differentiation by promoting cell proliferation, as indicated by lower cardiac differentiation potential of S stage mesoderm cells in BAF250a knockout cells. Moreover, we find that BAF250a is also required for proper recruitment of OCT4/β-CATENIN to CCND2/3 promoters in mesoderm cells. Thus, our results showed that BAF250a coordinates Oct4/β-CATENIN to regulate the chromatin structure in both differentiation and proliferation genes and therefore control the balance of cell cycle exit and cardiac differentiation.

In summary, we demonstrate that the physical interaction of BAF250 with Oct4 and WNT/β-CATENIN complex is critical for cardiac differentiation by directly regulating cardiac mesodermal lineage gene expression and cell cycle phases. Our study also indicates that manipulating the cell cycle state of mesoderm cell may further enhance the cardiac lineage specification. Further studies in understanding how BAF complex interplays with developmental signals in maintaining proliferation and differentiation balance may provide key molecular mechanisms for therapies against heart disease.

## Data Availability Statement

The raw data supporting the conclusions of this article will be made available by the authors, without undue reservation, to any qualified researcher.

## Author Contributions

IL, YZ, and ZW designed the study and wrote the manuscript. IL, ST, and VC performed the experiments. ZW gave final approval of manuscript.

## Conflict of Interest

The authors declare that the research was conducted in the absence of any commercial or financial relationships that could be construed as a potential conflict of interest.
